# Supplementary material for: The nonlinear association between triglyceride glucose-body mass index and femoral neck BMD in nondiabetic elderly men: NHANES 2005-March 2020
Source: PLoS One. 2024 Jan 23;19(1):e0296935. doi: 10.1371/journal.pone.0296935 (PMC10805317; doi:10.1371/journal.pone.0296935)
Supplement: S1 Table — (DOCX) [file pone.0296935.s001.docx]

Supplementary Table 1 Univariate and multivariate eﬀects of the association between TyG index on FN BMD (mg/cm^2^) in non-diabetic elderly men in NHANES.

|  | Crude model | | | Multivariate-adjusted Model 1 | | Multivariate-adjusted Model 2 | | | |
| --- | --- | --- | --- | --- | --- | --- | --- | --- | --- |
|  | β (95% CI) | | P-value | β (95% CI) | P-value | β (95% CI) | | P-value | |
| TyG-BMI | | |  |  |  |  |  |  | |
| per SD increase (continued) | | | 0.267 (-0.500, 1.033) | 0.495 | 0.041 (-0.708, 0.790) | 0.915 | 3.325 (0.041, 6.609) | 0.048 | |
| TyG-BMI tertile | | |  |  |  |  |  |  | |
| T1: < 8.25 (low) | | | Ref. |  | Ref. |  | Ref. |  | |
| T2: 8.26-8.72 (intermediate) | | | -0.232 (-2.109, 1.646) | 0.809 | -0.182 (-2.012, 1.647) | 0.845 | -1.198 (-4.323, 1.927) | 0.453 | |
| T3: ≥ 8.73 (high) | | | 0.235 (-1.641, 2.111) | 0.806 | -0.221 (-2.053, 1.611) | 0.813 | 0.269 (-4.493, 5.030) | 0.912 | |

NOTs:

Crude model: None
Multivariate-adjusted Model 1: age;

Multivariate-adjusted Model 2: age; race/ethnicity; education; marital status; drinking status; smoking status; SBP (Smooth); DBP (Smooth); TC; HDL; LDL; family of osteoporosis; physical activity; PIR; serum calcium; serum phosphorus; serum 25(OH)D_3_.

BMD, bone mineral density; BMI, body mass index; DBP, diastolic blood pressure; FN BMD, Femoral Neck BMD; HDL, high-density lipoprotein cholesterol; LD, low-density lipoprotein cholesterol; PIR, family income to poverty ratio; SBP, systolic blood pressure; TC, Total cholesterol; TyG-BMI, triglyceride and glucose- body mass index.
