# Supplementary material for: The nonlinear association between triglyceride glucose-body mass index and femoral neck BMD in nondiabetic elderly men: NHANES 2005-March 2020
Source: PLoS One. 2024 Jan 23;19(1):e0296935. doi: 10.1371/journal.pone.0296935 (PMC10805317; doi:10.1371/journal.pone.0296935)
Supplement: Attachment — Submitted filename: Response to Reviewer.docx [file pone.0296935.s003.docx]

Dear editors:

Thank you very much for your letter dated 04-Aug-2023. I am sorry that some problems in my computer lead to some errors in submitting the manuscript. Based on your comment and request, we have made extensive modification on the original manuscript. Here, we attached revised manuscript in the formats of both PDF and MS word, for your approval. A document answering every question from the referees was also summarized and enclosed.
A revised manuscript (PONE-D-22-30746) with the correction sections yellow marked was attached as the supplemental material and for easy check/editing purpose.

Should you have any questions, please contact us without hesitate.

Here below is our description on revision according to the letter.

1. Please ensure that your manuscript meets PLOS ONE's style requirements, including those for file naming. The PLOS ONE style templates can be found at https://journals.plos.org/plosone/s/file?id=wjVg/PLOSOne_formatting_sample_main_body.pdf and https://journals.plos.org/plosone/s/file?id=ba62/PLOSOne_formatting_ sample_title_authors_affiliations.pdf

Reply:

Done accordingly.

2. "PLOS requires an ORCID iD for the corresponding author in Editorial Manager on papers submitted after December 6th, 2016. Please ensure that you have an ORCID iD and that it is validated in Editorial Manager. To do this, go to ‘Update my Information’ (in the upper left-hand corner of the main menu), and click on the Fetch/Validate link next to the ORCID field. This will take you to the ORCID site and allow you to create a new iD or authenticate a pre-existing iD in Editorial Manager. Please see the following video for instructions on linking an ORCID iD to your Editorial Manager account: https://www.youtube.com/watch?v=_xcclfuvtxQ"

Reply:

ORCID ID for the corresponding author (Chenghu Huang) is 0000-0001-7088-1753.

3. For studies reporting research involving human participants, PLOS ONE requires authors to confirm that this specific study was reviewed and approved by an institutional review board (ethics committee) before the study began. Please provide the specific name of the ethics committee/IRB that approved your study, or explain why you did not seek approval in this case.
Once you have amended this/these statement(s) in the Methods section of the manuscript, please add the same text to the “Ethics Statement” field of the submission form (via “Edit Submission”).

For additional information about PLOS ONE ethical requirements for human subjects research, please refer to http://journals.plos.org/plosone/s/submission-guidelines#loc-human-subjects-research.

Reply:

We already mentioned the ethics statement on Page 4, Lines 116-118. “Human Research Ethics Committee approval was not needed, as all data used in the analysis were deidentified and made publicly available.”

Response to Reviewer 1:

1. Abstracts
1) Abstract should be informative, background did not explain the question of this research and the answer which authors search for it

Reply: We explained the question on Page 2, Lines 31-34.
2) Please indicate an exact explanation of TyG-BMI

Reply: We explained the TyG-BMI on Page 2, Lines 31-34.
3) Keywords: are these keywords are Mesh terms? Word that serves as a keyword, as to the meaning of that condition must be a Mesh term
Reply: We corrected the keywords on Page 2, Lines 54-55.

2. Introduction
-“Multiple US and international organizations recommend that clinicians consider BMD at the femoral neck (FN) to diagnose osteoporosis and low bone mass”
please expand and explain more about using FN as gold standard to diagnose osteoporosis and low bone mass
Reply: We explained the confusions on Page 3, Lines 60-63 and Lines 66-69.

3. -Please correct :
 Line 68: Whether insulin resistance (IR) can a-ect bone remain unclear. In experimental
Reply: We corrected it on Page 3, Lines 77-78.

4. Results
It would be great if the authors prepare a correlation diagram for the study

Reply: Pearson's correlation coefficient between the TyG-BMI and FN BMD was 0.321 (P < 0.001). We analyzed this on Page 9, Lines 235-236.

5. Discussion
- The authors should list and shortly discuss the limitation of their study, for instance their limited number of participants and small sample size

Reply: We listed the limitation of our study on Page 13, Lines 346-348.

Response to Reviewer 2:

Reviewer #2: The authors aimed to correlate the triglyceride glucose-body mass index (TyG-BMI), a novel marker for assessing IR with femoral neck (FN) BMD in nondiabetic elderly men. they used data from the National Health and Nutrition Examination Survey (NHANES) database. they results indicated TyG-BMI is positively associated with FN BMD in a nonlinear fashion among elderly men without diabetes. The study is well designed, the statistical modeling supports the results.
minor:
1. Please consider all abbreviation in text and title and abstract. May some abbreviations were used without mentioning the full expression for the first time. Also check them for tables and graphs too.

Reply:

We correct those errors.
2. I believe discussion would be more extended according to the results. I feel this section did not bring all findings under debate. For example, the results based of baseline indicated there would be potentially groups under risk of more or less association for TyG-BMI (for instance according to etnicity,...).

Reply:

We extensively discussed the results on Page 12, Lines 320-325. “We also confirmed that the nonlinear positive correlation between TyG-BMI on FN BMD persisted in nondiabetic elderly men. In the baseline, some variables (such as the ratio of race/ethnicity) varied with the levels of TyG-BMI (Table 1). However, in almost all subgroups, the correlation between TyG-BMI and FN BMD was nonlinear and positive, with no significant interaction eﬀect in the crude model and multivariate-adjusted models (Figure 3).

Response to Reviewer 3:

1. This study analyzes the relationship between TYG-BMI and FN BMD, and assumes that it is a new indicator. Therefore, it is suggested to analyze the relationship between TYG and FN BMD at the same time and compare them.

Reply: We have already focused on the relationship between TyG and FN BMD. However, because the relationship between them is ambiguous, we did not mention it in the manuscript. In this revised manuscript, I have added this on Page 11, Lines 301-304.

2. Why only elderly men are included in the study and women are excluded is not clearly explained in the introduction and background.

Reply: We clearly explained it in the introduction and background Page 4, Lines 95-96.

4. The full text analysis strategy needs to be further reorganized, and the relationship between TYG-BMI and FN BMD needs to be analyzed qualitatively and quantitatively.

Reply: We quantitatively discussed it on Page 11, Lines 312-316 and qualitatively on Page 12, Lines 319-324.

Many grammatical or typographical errors have been revised.

All the lines and pages indicated above are in the revised manuscript.

Thank you and all the reviewers for the kind advice.

Yours sincerely,
Chenghu Huang
E-mail: tigerhchh@126.com
